# Lost in Translation: Barriers in Psychiatric Care for Patients With Communication Impairments

**DOI:** 10.7759/cureus.110389

**Published:** 2026-06-07

**Authors:** Emily D Duckworth, Tasmia S Khan, Diego Regalado, Oluwole A Babatunde

**Affiliations:** 1 Department of Biomedical Sciences, University of South Carolina School of Medicine Greenville, Greenville, USA; 2 Department of Psychiatry, Prisma Health, Greer, USA

**Keywords:** aphasia, communication impairment, interdisciplinary care, psychiatric assessment, stroke

## Abstract

Effective communication is central to psychiatric evaluation and treatment. Patients with acquired communication impairments, such as post-stroke aphasia, are at increased risk for misdiagnosis, delayed care, and suboptimal treatment. We aim to better understand the unique needs of psychiatric patients with communication difficulties, common pitfalls in their care, and ways to improve clinical assessment, diagnosis, and treatment of their mental health conditions.

We present the case of a 49-year-old woman with a history of major depressive disorder, generalized anxiety disorder, post-traumatic stress disorder, functional neurological symptom disorder, and a left middle cerebral artery ischemic stroke resulting in non-fluent aphasia. She was admitted to an inpatient psychiatric unit for suicidal ideation in the context of significant post-stroke functional decline. Her hospitalization was complicated by limited verbal communication and reliance on nonverbal modalities, cognitive fatigue related to communicating, persistent depression symptoms, as well as fluctuating reports of perceptual disturbances. Communication barriers significantly impacted assessment of mood, suicidality, and perceptual symptoms, contributing to diagnostic uncertainty and complex medical decision-making.

This case highlights how expressive aphasia can obscure psychiatric assessment, increase reliance on interpretation by clinicians and caregivers, and contribute to potential misunderstanding of symptoms. It also underscores the importance of multimodal communication strategies and interdisciplinary collaboration.

Psychiatric patients with communication impairments require tailored assessment approaches to reduce diagnostic error, gain greater understanding of the patient's clinical presentation, and improve overall patient care. Increased awareness and structured communication strategies may mitigate disparities in this vulnerable population.

## Introduction

Effective communication is fundamental to psychiatric assessment, diagnosis, and treatment, relying heavily on patient-reported symptoms, nuanced emotional expression, and longitudinal narrative coherence [1]. A subset of psychiatric patients, however, experience significant communication barriers that complicate clinical interactions and compromise quality of care [2,3]. These barriers occur across diverse populations, including individuals who are deaf or hard of hearing, as well as patients who have acquired communication impairments such as aphasia following neurological injury from stroke. Congenital or acquired communication impairments can also exacerbate existing language and cultural barriers. When communication is limited, clinicians may misinterpret symptoms, delay diagnosis, or fail to engage patients in meaningful therapeutic dialogue, perpetuating disparities in mental health outcomes in these marginalized populations [4-6].

Aphasia and similar deficits disrupt patients’ ability to express emotions, describe experiences, and engage with clinicians verbally or in writing. Non-fluent aphasia impairs the ability to produce language while often preserving comprehension [7]. This mismatch can lead to underestimation of patient insight, misinterpretation of affect, and difficulty eliciting key psychiatric symptoms such as suicidal ideation or perceptual disturbances. These challenges are compounded in patients with pre-existing psychiatric illness, where diagnostic clarity depends on detailed subjective reporting. Furthermore, these deficits are associated with increased risk of depression, anxiety, and social isolation [8,9]. Stroke patients face pervasive barriers in accessing psychological support, including clinicians’ feelings of being unprepared for communication breakdowns and the lack of structured support systems tailored to aphasic patients within healthcare environments [2].

Psychiatric populations with communication difficulties are at increased risk for substandard outcomes due to both individual language impairments and systemic healthcare barriers driving social determinants of health [10]. These interconnected issues demand targeted research that makes clear the unique needs of post-stroke patients within psychiatric contexts. Consequently, these issues inform appropriate practices in mental health care that help mitigate communication difficulties. We present a case of a patient with post-stroke expressive aphasia whose inpatient psychiatric care was significantly impacted by communication limitations, highlighting diagnostic challenges, treatment considerations, and opportunities for improving care.

## Case presentation

Patient history

The patient provided written informed consent for the publication of this case report. The patient was a 49-year-old woman with a complex psychiatric and neurologic history, including post-traumatic stress disorder, generalized anxiety disorder, and recurrent major depressive disorder. She also has a history of functional neurologic symptom disorder and seizure-like episodes previously characterized as a combination of epileptic and psychogenic nonepileptic events. In September 2024, she experienced a left middle cerebral artery ischemic stroke requiring mechanical thrombectomy. Prior to her stroke, the patient lived alone and was able to engage in activities of daily living independently. She was enrolled in school and was active in multiple areas of her life. Following the stroke, she developed persistent expressive aphasia, dysarthria, and right-sided weakness. Her aphasia was characterized by intact comprehension but markedly impaired speech production, consistent with non-fluent aphasia. Notably, her ability to participate in psychiatric care became severely restricted due to her aphasia, requiring alternative communication strategies. Following her stroke, the patient experienced a significant decline in functional independence, including an inability to drive and increased reliance on family support. Communication strategies included the use of flash cards with images and words, use of emojis, physical gesturing, writing, typing, and having her sister speak on her behalf.

Inpatient psychiatric admission

In January 2026, the patient presented to her outpatient psychiatrist endorsing suicidal ideation with intent and plan. She was subsequently admitted to an inpatient psychiatric unit. Her suicidal ideation was attributed to worsening depression in the context of post-stroke disability, loss of independence, and psychosocial stressors, including feeling burdensome to her family caretakers.

At admission, communication was significantly limited. The patient relied on physical gestures, nodding, emojis on a tablet, and yes/no responses. She was described as a “somewhat reliable historian”; however, this was not due to suspected deception, withholding, or intellectual disability. Rather, this distinction reflected the inherent uncertainty in symptom reporting due to communication constraints and clinicians’ inability to know whether they had accurately interpreted the patient’s communications.

During her 21-day hospitalization, clinicians frequently relied on simplified and focused questioning, observation of affect, and collateral information from her outpatient psychiatrist. The patient’s outpatient psychiatrist was routinely contacted and provided ample collateral to supplement limited structured interviewing. The patient had seen her outpatient psychiatrist for multiple years prior to her stroke, and he knew her history well. Assessment of mood and suicidality was variable, and her choice of mode of communication also changed throughout her admission. The patient conveyed emotions using gestures, emojis, and limited verbalizations, with descriptions ranging from “so-so” to expressions of sadness and frustration. Team members would ask confirmatory follow-up questions to ensure they were accurately interpreting her gestures or emoji use, and the patient would reply with head nodding or further clarification. Though daily fluctuations were observed, her mood and anxiety gradually improved throughout her stay, and she eventually took an active role in her discharge planning and placement identification. Suicidal ideation fluctuated throughout admission, at times including passive thoughts and at others, more specific plans such as walking into traffic. The inability to obtain nuanced verbal descriptions complicated risk assessment and contributed to ongoing diagnostic uncertainty regarding the severity and trajectory of her depression. Furthermore, the patient was visibly fatigued by prolonged communication, so interviews were limited in duration and detail. Both the length of communication and the array of communication modalities utilized by the patient throughout a single conversation increased the cognitive load for the patient and added to her communication burdens. Throughout her hospital stay, clinical teams became more comfortable and familiar with her methods of communication and readily adapted to her preferences. Once the patient felt she had adequate accommodations and time to express herself, this improved communication between the provider and patient. The patient was active and engaged in group therapy and milieu activities throughout the course of her inpatient stay. However, she did not often attempt to speak with other patients; instead, she primarily interacted physically. 

During admission, the patient reported tactile and visual hallucinations described as “fairies,” as well as intermittent auditory phenomena such as whispering. These reports prompted concern for psychosis, leading to initiation of low-dose risperidone. Characterization of these experiences was limited by communication barriers, and details regarding her perceptual disturbances were difficult to elicit. Over time, perceived inconsistencies in her experience of these hallucinations emerged, including descriptions suggesting non-distressing, transient, or physiologically triggered phenomena due to positional changes.

Pharmacologic management was complex due to diagnostic uncertainty, comorbid medical conditions, and concern for polypharmacy. Venlafaxine was increased to target depressive symptoms, her home buspirone was continued, and risperidone was initiated for suspected psychosis. Consideration of lithium was deferred due to medical risks and drug interactions. Medication decisions were further complicated by limited patient ability to report side effects and symptom changes.

During the patient’s hospitalization, she experienced progressively worsening generalized abdominal discomfort. Further questioning prompted by the severity of her symptoms revealed that she had not had a bowel movement for 14 days. The patient’s constipation was likely multifactorial in origin, including medication side effects (notably risperidone), reduced mobility, and neurologic impairment. Her symptoms required escalation of bowel regimens and eventual transfer to the emergency department for management.

Post-discharge course

Post-discharge outpatient follow-up with her psychiatrist suggested that the reported "fairies" were more likely visual phenomena associated with positional changes or stress, rather than true hallucinations indicative of psychosis. This reinterpretation led to discontinuation of antipsychotic therapy. Although she continued to experience chronic suicidal ideation, she identified protective factors, including family relationships and personal interests. She demonstrates overall psychiatric stabilization and continues monthly follow-up with her outpatient psychiatrist. Since her stroke, psychiatric appointments have required approximately twice the usual amount of time to allow for communication, even when interviews are highly focused and abbreviated. Despite these extended visits, clinical encounters often permit only essential safety assessments and basic information gathering, while more detailed exploration of symptoms, experiences, and clinical nuances would require substantially more time.

## Discussion

This case illustrates the profound impact communication impairments can have on psychiatric care. Accurate psychiatric diagnosis depends heavily on patients' ability to describe subjective experiences through open-ended dialogue, emotional narratives, and nuanced symptom reporting [11]. In patients with post-stroke aphasia, however, this process becomes considerably more challenging. Consistent with prior literature describing barriers to mental health care among individuals with aphasia and other communication disabilities, our patient's expressive language deficits limited her ability to convey symptoms, elaborate on experiences, and participate fully in diagnostic interviews [2,5,6,12]. 

The diagnostic uncertainty encountered during this hospitalization illustrates many of the challenges described in previous studies. The patient's reported experiences of "fairies," whispering, and tactile sensations were initially interpreted as possible psychotic symptoms, leading to treatment with risperidone. Further longitudinal assessment after discharge suggested that these experiences were more consistent with transient physiologic or stress-related perceptual phenomena than true psychosis. Similar concerns have been reported in communication-impaired populations, where clinicians may be forced to rely on fragmented patient reports, behavioral observations, or collateral information when direct symptom characterization is limited [13-17]. Such circumstances increase the risk of diagnostic overshadowing, symptom misclassification, and both under- and over-treatment. In this case, communication barriers restricted the patient's ability to provide the detailed contextual information necessary to distinguish psychotic from non-psychotic experiences, contributing to diagnostic ambiguity. 

The patient's hospitalization also demonstrated how communication barriers can influence treatment decisions and patient safety. Psychiatric management depends heavily on ongoing patient feedback; therefore, difficulty assessing symptom progression and medication effects complicated pharmacologic decision-making throughout admission. The delayed recognition of severe constipation, ultimately requiring emergency department evaluation, highlights how communication limitations may hinder identification of adverse effects and other medical concerns. Adults with communication disabilities experience poorer healthcare outcomes overall, in part because symptoms may go unrecognized or be identified later in their course [12]. Our patient's inability to easily communicate evolving physical discomfort likely contributed to delays in recognizing the severity of her condition. This experience suggests that clinicians caring for patients with significant communication impairments should consider supplementing subjective symptom assessment with more structured monitoring, frequent reassessment, and increased attention to objective clinical findings. 

An important observation from this case was the absence of a formal assessment of the patient's communication preferences upon admission. Although the patient arrived with an iPad equipped with communication software (Proloquo2Go), clinicians initially assumed this was her preferred method of communication. Over time, it became apparent that prolonged use of the device was cognitively demanding and frustrating for her. She often preferred simpler strategies such as yes/no questioning, gesturing, emojis, and written responses. Notably, her outpatient psychiatrist later discovered that she could communicate effectively through short written sentences, a modality that was underutilized during hospitalization. These observations closely mirror findings from Morris et al., who reported discrepancies between the communication methods patients with aphasia prefer and those physicians commonly employ [12]. Our case reinforces the importance of formally identifying communication preferences early in care and providing patients with multiple communication options rather than assuming a single modality will be effective. 

The literature further supports several interventions that may improve psychiatric care for patients with communication impairments. Studies involving aphasic, deaf, and hard-of-hearing populations emphasize the importance of providing preferred communication modalities, allowing additional response time, minimizing interruptions, and incorporating caregivers or other communication partners when appropriate [13-19]. Many of these principles proved relevant in the present case. Communication improved as clinicians became more familiar with the patient's preferred methods, adapted their interview style, and collaborated closely with her outpatient psychiatrist. Similarly, caregiver involvement and collateral information were critical for understanding symptom chronology and maintaining diagnostic continuity. These findings align with evidence supporting interdisciplinary and caregiver-supported approaches for individuals with communication disabilities [19]. 

This case underscores the importance of maintaining diagnostic flexibility and avoiding premature conclusions when evaluating psychiatric symptoms in communication-impaired patients. Since psychiatric diagnoses often depend on nuanced subjective reports, clinicians may be particularly vulnerable to cognitive biases when communication is limited. Repeated assessments, longitudinal observation, and ongoing reassessment of diagnostic formulations may therefore be especially important in this population [18]. The eventual reinterpretation of the patient's perceptual experiences following discharge illustrates how diagnoses formed under conditions of limited communication may evolve as additional information becomes available. This case adds to a growing body of literature demonstrating that communication-impaired individuals represent a vulnerable population at increased risk for healthcare disparities, diagnostic error, and suboptimal treatment outcomes [10,13]. Greater attention to communication assessment, multimodal communication strategies, interdisciplinary collaboration, and longitudinal reassessment may help reduce these risks. Future work should focus on developing standardized approaches for psychiatric evaluation in patients with significant communication impairments and strengthening collaboration between psychiatric, neurologic, speech-language pathology, and rehabilitation services.

## Conclusions

Communication impairments, particularly non-fluent aphasia following stroke, can significantly complicate psychiatric assessment, diagnosis, and treatment. This case demonstrates how limited communication may lead to diagnostic uncertainty, misinterpretation of symptoms, and complex clinical decision-making. Adopting structured, multimodal communication strategies and maintaining diagnostic flexibility are essential to improving care for this population. Greater provider awareness, targeted interventions and accommodations, coordination of care, and increased patient education may help reduce disparities and improve outcomes in psychiatric patients with communication difficulties.
